# Supplementary material for: Gender Disparities of Heart Disease and the Association with Smoking and Drinking Behavior among Middle-Aged and Older Adults, a Cross-Sectional Study of Data from the US Health and Retirement Study and the China Health and Retirement Longitudinal Study
Source: Int J Environ Res Public Health. 2022 Feb 15;19(4):2188. doi: 10.3390/ijerph19042188 (PMC8872153; doi:10.3390/ijerph19042188)
Supplement: Supplementary file 1 [file ijerph-19-02188-s001.zip › ijerph-1541734-supplementary.pdf]

Gender disparities of heart disease and the association with smoking and drinking behavior among middle-aged and older adults, a cross-sectional study of data from the US Health and Retirement Study and the China Health and Retirement Longitudinal Study

Supplementary File

Table of contents

Table S1. Sample sizes (N) and weighted percentages (wt%) of smoking and drinking behavior by age group for men and women in the US and China. ……………………………………………………………………………………………….… Page 2

Table S2. Weighted prevalence (Prev) of heart disease with 95% CIs for men and women in the US and China. …………….. Page 3

Table S3. Crude prevalence ratio PR (cPR) of heart disease with 95% CIs for men and women in the US and China. ……….. Page 6

Table S4. Prevalence ratio (adjPR) of heart disease with 95% CIs under the adjustment of age group, education level, household tertiles, high blood pressure, diabetes, sleep quality, BMI categories and US race/ethnicity for men and women in the US and China ……………..……………. ……………. ……………..…………….…………….……………..…………… Page 8

Table S1. Sample sizes (N) and weighted percentages (wt%) of smoking and drinking behavior by age group for men and women in the US and China.

	Neither<br>N (wt%)	Only drinking<br>N (wt%)	Only smoking<br>N (wt%)	Both<br>N (wt%)
US Men				
50-59	350 (11.3)	937 (35.5)	410 (12.0)	1283 (41.2)
60-69	317 (12.2)	605 (28.5)	513 (18.8)	1057 (40.4)
70-79	261 (14.6)	283 (17.2)	468 (26.4)	708 (41.8)
80+	223 (17.7)	194 (17.5)	359 (27.2)	427 (37.6)
US Women				
50-59	762 (15.8)	1120 (31.7)	618 (15.4)	1368 (37.2)
60-69	895 (23.6)	836 (28.7)	661 (17.0)	971 (30.7)
70-79	796 (30.7)	443 (19.9)	577 (21.8)	616 (27.6)
80+	751 (39.9)	304 (16.6)	427 (21.3)	392 (22.1)
China Men				
50-59	153 (5.3)	305 (11.1)	561 (20.7)	1676 (62.9)
60-69	139 (5.2)	272 (9.8)	564 (21.0)	1696 (64.0)
70-79	120 (9.5)	120 (9.7)	324 (25.0)	729 (55.8)
80+	37 (20.4)	35 (7.9)	89 (23.8)	179 (47.9)
China Women				
50-59	2210 (74.7)	537 (19.2)	135 (4.0)	64 (2.1)
60-69	1925 (70.9)	545 (19.3)	195 (7.0)	82 (2.8)
70-79	814 (66.8)	223 (18.9)	127 (10.3)	55 (4.0)
80+	275 (62.0)	75 (20.0)	48 (12.2)	20 (5.8)

Table S2. Weighted prevalence (Prev) of heart disease with 95% CIs for men and women in the US and China.

Variables	US Men		US Women		China Men		China Women	
	Prev (95% CI)	*p*	Prev (95% CI)	*p*	Prev (95% CI)	*p*	Prev (95% CI)	*p*
Overall	24.5 (22.5,26.6)		20.6 (19.3,22.1)		16.1 (15.1,17.2)		22.9 (21.7,24.1)	
Age group (years)		<0.001		<0.001		<0.001		<0.001
50-59	12.9 (11.4,14.5)		11.3 (9.9,12.8)		10.1 (8.9,11.4)		17.1 (15.3,18.9)	
60-69	23.4 (21.3,25.6)		18.9 (16.9,21.1)		17.5 (15.7,19.4)		25.4 (23.6,27.2)	
70-79	36.4 (33.8,39.2)		28.0 (26.2,30.0)		24.5 (22.0,27.3)		30.6 (27.4,34.0)	
80+	53.0 (49.8,56.2)		39.7 (37.5,41.9)		21.6 (16.2,28.3)		25.4 (20.7,30.8)	
Smoking and drinking behavior		<0.001		<0.001		0.933		<0.001
Neither	27.4 (23.5,31.7)		23.5 (21.3,25.8)		15.4 (11.8,19.8)		21.7 (20.4,23.0)	
Only drinking	15.4 (13.3,17.7)		14.8 (12.7,17.2)		16.7 (14.0,19.8)		21.8 (18.6,25.5)	
Only smoking	35.5 (32.4,38.6)		31.5 (28.7,34.4)		16.6 (14.1,19.4)		34.4 (30.0,39.2)	
Both	24.9 (22.6,27.3)		17.2 (15.3,19.2)		16.0 (14.8,17.3)		34.2 (27.7,41.4)	
Education		0.051		<0.001		0.001		0.024
Less than high school	26.9 (23.2,31.0)		28.3 (25.3,31.6)		15.5 (14.5,16.6)		22.4 (21.2,23.7)	
High school or equivalent	25.4 (22.9,28.1)		23.1 (21.3,25.0)		17.2 (13.7,21.3)		29.1 (25.0,33.6)	
Associate degree or higher	22.6 (20.1,25.2)		14.4 (12.7,16.3)		27.8 (21.8,34.7)		23.5 (13.4,37.8)	
Household income		<0.001		<0.001		0.024		0.154
Tertile 1	30.2 (26.9,33.7)		28.0 (26.1,29.9)		14.5 (12.7,16.6)		23.2 (21.0,25.5)	
Tertile 2	29.1 (26.1,32.3)		22.4 (20.2,24.8)		16.8 (14.8,18.9)		24.0 (21.8,26.4)	
Tertile 3	19.0 (17.1,21.1)		13.4 (11.9,15.1)		18.7 (16.6,20.9)		24.5 (21.5,27.8)	
Did not report	-		-		15.1 (13.3,17.1)		21.2 (19.4,23.2)	
High blood pressure		<0.001		<0.001		<0.001		<0.001
No	14.0 (12.3,15.9)		11.6 (10.5,12.9)		10.5 (9.4,11.7)		15.5 (14.2,16.8)	
Yes	32.3 (30.1,34.6)		28.1 (26.4,29.9)		26.4 (24.3,28.6)		35.0 (32.8,37.2)	
Diabetes		<0.001		<0.001		<0.001		<0.001
No	20.7 (18.8,22.8)		17.9 (16.5,19.2)		14.5 (13.5,15.6)		20.7 (19.5,22.0)	
Yes	35.8 (33.0,38.8)		30.2 (27.8,32.8)		31.6 (27.1,36.4)		38.8 (35.2,42.5)	
Sleep quality		<0.001		<0.001		0.009		<0.001
Restless	33.2 (29.9,36.7)		25.8 (23.6,28.1)		18.8 (16.8,21.0)		26.7 (24.8,28.6)	

Good	22.0 (20.0,24.2)		18.8 (17.4,20.3)		15.2 (14.0,16.4)		20.1 (18.6,21.8)	
Did not report	40.5 (25.9,56.9)		36.8 (24.7,50.8)		16.7 (13.0,21.2)		22.9 (18.3,28.3)	
BMI		0.047		0.139		<0.001		<0.001
Normal	24.4 (21.1,28.2)		19.7 (17.9,21.6)		14.4 (13.2,15.7)		19.2 (17.6,20.9)	
Overweight	22.6 (20.4,24.9)		19.7 (18.0,21.6)		19.5 (17.3,21.9)		26.6 (24.2,29.2)	
Obese	26.6 (23.8,29.6)		22.3 (20.3,24.5)		30.9 (24.1,38.7)		33.5 (28.7,38.6)	
Underweight	34.9 (22.2,50.2)		20.0 (15.3,25.7)		16.3 (12.7,20.6)		21.2 (17.0,26.0)	
Did not report	20.3 (7.2,45.3)		19.5 (12.4,29.2)		15.1 (12.6,18.0)		24.8 (21.9,27.9)	
Race/ethnicity		<0.001		<0.001				
White	26.6 (24.4,29.0)		21.2 (19.8,22.8)		-		-	
Hispanic	17.1 (14.2,20.4)		15.8 (12.9,19.1)		-		-	
Black	20.2 (17.6,23.0)		23.4 (20.4,26.6)		-		-	
Other	17.9 (14.1,22.6)		15.6 (12.1,19.8)		-		-	

Table S3. Crude prevalence ratio PR (cPR) of heart disease with 95% CIs for men and women in the US and China.

Variables	US Men		US Women		China Men		China Women	
	cPR (95% CI)	*p*	cPR (95% CI)	*p*	cPR (95% CI)	*p*	cPR (95% CI)	*p*
Age group (years)								
50-59	Ref		Ref		Ref		Ref	
60-69	1.81 (1.56, 2.10)	<0.001	1.68 (1.42, 1.99)	<0.001	1.73 (1.47, 2.03)	<0.001	1.49 (1.31, 1.69)	<0.001
70-79	2.82 (2.45, 3.25)	<0.001	2.49 (2.16, 2.88)	<0.001	2.43 (2.07, 2.85)	<0.001	1.79 (1.54, 2.09)	<0.001
80+	4.11 (3.62, 4.66)	<0.001	3.53 (3.06, 4.07)	<0.001	2.14 (1.58, 2.91)	<0.001	1.49 (1.19, 1.87)	<0.001
Smoking and drinking behavior								
Neither	Ref		Ref		Ref		Ref	
Only drinking	0.56 (0.47, 0.67)	<0.001	0.63 (0.53, 0.75)	<0.001	1.09 (0.80, 1.48)	0.604	1.01 (0.85, 1.19)	0.920
Only smoking	1.29 (1.11, 1.51)	0.002	1.34 (1.20, 1.51)	<0.001	1.08 (0.80, 1.46)	0.627	1.59 (1.37, 1.84)	<0.001
Both	0.91 (0.78, 1.06)	0.210	0.73 (0.64, 0.84)	<0.001	1.04 (0.79, 1.36)	0.775	1.58 (1.28, 1.95)	<0.001
Education								
Less than high school	Ref		Ref		Ref		Ref	
High school or equivalent	0.94 (0.82, 1.08)	0.412	0.82 (0.71, 0.94)	0.005	1.11 (0.88, 1.39)	0.385	1.30 (1.11, 1.52)	0.001
Associate degree or higher	0.84 (0.71, 0.99)	0.035	0.51 (0.43, 0.60)	<0.001	1.79 (1.41, 2.28)	<0.001	1.05 (0.62, 1.76)	0.865
Household income								
Tertile 1	Ref		Ref		Ref		Ref	
Tertile 2	0.96 (0.85, 1.09)	0.557	0.80 (0.72, 0.89)	<0.001	1.15 (0.96, 1.38)	0.121	1.04 (0.90, 1.19)	0.608
Tertile 3	0.63 (0.55, 0.72)	<0.001	0.48 (0.42, 0.54)	<0.001	1.29 (1.07, 1.54)	0.006	1.06 (0.90, 1.24)	0.476
Did not report	-		-		1.04 (0.87, 1.25)	0.657	0.92 (0.80, 1.05)	0.193
High blood pressure								
No	Ref		Ref		Ref		Ref	
Yes	2.31 (2.08, 2.57)	<0.001	2.42 (2.18, 2.69)	<0.001	2.52 (2.20, 2.89)	<0.001	2.26 (2.03, 2.51)	<0.001
Diabetes								
No	Ref		Ref		Ref		Ref	
Yes	1.73 (1.58, 1.89)	<0.001	1.69 (1.55, 1.84)	<0.001	2.18 (1.85, 2.57)	<0.001	1.87 (1.67, 2.09)	<0.001
Sleep quality								
Restless	Ref		Ref		Ref		Ref	
Good	0.66 (0.59, 0.74)	<0.001	0.73 (0.66, 0.80)	<0.001	0.81 (0.70, 0.92)	0.002	0.75 (0.68, 0.84)	<0.001
Did not report	1.22 (0.80, 1.85)	0.355	1.43 (1.00, 2.03)	0.047	0.89 (0.68, 1.16)	0.384	0.86 (0.68, 1.08)	0.191

BMI								
Normal	Ref		Ref		Ref		Ref	
Overweight	0.92 (0.79, 1.08)	0.315	1.00 (0.90, 1.12)	0.947	1.35 (1.17, 1.57)	<0.001	1.39 (1.22, 1.58)	<0.001
Obese	1.09 (0.94, 1.27)	0.259	1.14 (1.03, 1.26)	0.016	2.15 (1.67, 2.76)	<0.001	1.75 (1.47, 2.07)	<0.001
Underweight	1.43 (0.97, 2.09)	0.067	1.02 (0.77, 1.34)	0.895	1.13 (0.88, 1.46)	0.344	1.11 (0.88, 1.39)	0.389
Did not report	0.83 (0.32, 2.12)	0.692	0.99 (0.64, 1.53)	0.965	1.05 (0.86, 1.28)	0.650	1.30 (1.12, 1.50)	<0.001
Race/ethnicity								
White	Ref		Ref					
Hispanic	0.64 (0.53, 0.77)	<0.001	0.74 (0.60, 0.92)	0.006	-		-	
Black	0.76 (0.66, 0.88)	<0.001	1.10 (0.97, 1.25)	0.129	-		-	
Other	0.67 (0.53, 0.86)	0.002	0.73 (0.58, 0.93)	0.012	-		-	

Table S4. Prevalence ratio (adjPR) of heart disease with 95% CIs under the adjustment of age group, education level, household tertiles, high blood pressure, diabetes, sleep quality, BMI categories and US race/ethnicity for men and women in the US and China.

Variables	US Men		US Women		China Men		China Women	
	**adjPR (95% CI)**	***p***	**adjPR (95% CI)**	***p***	**adjPR (95% CI)**	***p***	**adjPR (95% CI)**	***p***
Age group (years)								
50-59	Ref		Ref		Ref		Ref	
60-69	1.55 (1.33, 1.81)	<0.001	1.51 (1.27, 1.79)	<0.001	1.68 (1.40, 2.01)	<0.001	1.36 (1.19, 1.55)	<0.001
70-79	2.11 (1.84, 2.43)	<0.001	1.89 (1.63, 2.19)	<0.001	2.31 (1.95, 2.73)	<0.001	1.50 (1.30, 1.74)	<0.001
80+	3.04 (2.64, 3.49)	<0.001	2.51 (2.14, 2.96)	<0.001	1.96 (1.40, 2.74)	<0.001	1.31 (1.04, 1.65)	0.024
Smoking and drinking behavior								
Neither	Ref		Ref		Ref		Ref	
Only drinking	0.75 (0.63, 0.89)	0.001	0.92 (0.78, 1.09)	0.309	1.23 (0.87, 1.76)	0.246	1.01 (0.86, 1.18)	0.948
Only smoking	1.16 (1.01, 1.35)	0.046	1.34 (1.21, 1.49)	<0.001	1.35 (0.94, 1.95)	0.103	1.48 (1.29, 1.70)	<0.001
Both	1.02 (0.89, 1.16)	0.817	0.97 (0.85, 1.11)	0.675	1.26 (0.91, 1.74)	0.162	1.54 (1.25, 1.89)	<0.001
Education								
Less than high school	Ref		Ref		Ref		Ref	
High school or equivalent	1.05 (0.94, 1.19)	0.369	0.98 (0.88, 1.09)	0.712	1.24 (0.97, 1.59)	0.082	1.48 (1.26, 1.75)	<0.001
Associate degree or higher	1.10 (0.96, 1.27)	0.177	0.83 (0.72, 0.96)	0.012	1.64 (1.28, 2.09)	<0.001	1.24 (0.74, 2.08)	0.406
Household income								
Tertile 1	Ref		Ref		Ref		Ref	
Tertile 2	0.85 (0.76, 0.94)	0.003	0.92 (0.82, 1.03)	0.136	1.12 (0.94, 1.33)	0.205	1.04 (0.91, 1.18)	0.582
Tertile 3	0.76 (0.66, 0.87)	<0.001	0.79 (0.69, 0.91)	0.001	1.24 (1.03, 1.48)	0.02	1.08 (0.92, 1.26)	0.351
Did not report	-		-		0.99 (0.82, 1.18)	0.872	0.92 (0.81, 1.04)	0.201
High blood pressure								
No	Ref		Ref		Ref		Ref	
Yes	1.75 (1.56, 1.95)	<0.001	1.72 (1.52, 1.94)	<0.001	2.12 (1.85, 2.42)	<0.001	1.92 (1.72, 2.13)	<0.001
Diabetes								
No	Ref		Ref		Ref		Ref	
Yes	1.35 (1.24, 1.46)	<0.001	1.29 (1.18, 1.41)	<0.001	1.55 (1.32, 1.82)	<0.001	1.44 (1.30, 1.61)	<0.001
Sleep quality								
Restless	Ref		Ref		Ref		Ref	
Good	0.77 (0.69, 0.85)	<0.001	0.79 (0.72, 0.86)	<0.001	0.81 (0.71, 0.92)	0.002	0.78 (0.71, 0.87)	<0.001
Did not report	0.98 (0.64, 1.48)	0.912	1.00 (0.73, 1.36)	0.975	0.87 (0.65, 1.16)	0.331	0.85 (0.69, 1.04)	0.118

BMI								
Normal	Ref		Ref		Ref		Ref	
Overweight	1.00 (0.86, 1.15)	0.951	0.96 (0.85, 1.08)	0.482	1.19 (1.02, 1.37)	0.022	1.22 (1.08, 1.38)	0.001
Obese	1.10 (0.94, 1.28)	0.235	1.05 (0.93, 1.17)	0.431	1.69 (1.30, 2.19)	<0.001	1.40 (1.18, 1.66)	<0.001
Underweight	1.15 (0.78, 1.69)	0.483	0.78 (0.60, 1.00)	0.051	1.05 (0.82, 1.36)	0.684	1.07 (0.86, 1.32)	0.546
Did not report	1.12 (0.40, 3.11)	0.825	0.98 (0.63, 1.54)	0.944	0.97 (0.79, 1.20)	0.791	1.20 (1.04, 1.38)	0.014
Race/ethnicity								
White	Ref		Ref					
Hispanic	0.65 (0.55, 0.77)	<0.001	0.67 (0.57, 0.79)	<0.001	-		-	
Black	0.70 (0.61, 0.81)	<0.001	0.89 (0.81, 0.99)	0.032	-		-	
Other	0.86 (0.68, 1.09)	0.206	0.84 (0.67, 1.06)	0.137	-		-	
